# Nomogram for Predicting Hemorrhagic Transformation Risk in Acute Ischemic Stroke Patients With Atrial Fibrillation

**DOI:** 10.1111/cns.70402

**Published:** 2025-04-26

**Authors:** Jingjuan Chen, Mingyi Bao, Chengguo Zhang, Dong Pan, Yanting Chen, Yongteng Xu, Feng Zhou, Yamei Tang

**Affiliations:** ^1^ Department of Neurology First People's Hospital of Foshan Foshan China; ^2^ Department of Neurology Sun Yat‐Sen Memorial Hospital, Sun Yat‐Sen University Guangzhou China; ^3^ Department of Neurology, The Eighth Affiliated Hospital Sun Yat‐Sen University Shenzhen China; ^4^ Guangdong Provincial Key Laboratory of Malignant Tumor Epigenetics and Gene Regulation Sun Yat‐Sen Memorial Hospital, Sun Yat‐Sen University Guangzhou China; ^5^ Guangdong Province Key Laboratory of Brain Function and Disease, Zhongshan School of Medicine Sun Yat‐Sen University Guangzhou China

**Keywords:** acute ischemic stroke, atrial fibrillation, hemorrhagic transformation, LASSO regression, nomogram

## Abstract

**Background:**

Hemorrhagic transformation (HT) is a critical complication in acute ischemic stroke (AIS) patients with atrial fibrillation (AF) awaiting anticoagulation reinitiation. No reliable predictive model exists for assessing HT risk for these patients. Clinical decisions typically rely on NIHSS score and infarct size; however, other relevant risk factors remain insufficiently explored. This study aimed to develop and validate a predictive model for assessing the risk of HT in AIS patients with AF from stroke onset to anticoagulation therapy reinitiation.

**Methods:**

This retrospective study included AIS patients with AF from two comprehensive medical centers in China. The primary outcome was HT postinfarction confirmed with CT/MRI before anticoagulation reinitiation. Significant predictors were identified via LASSO regression in the training set, followed by multivariable logistic regression for developing a predictive model and generating the nomogram. Model performance was validated in a separate external cohort.

**Results:**

In the training cohort (*n* = 629), 174 patients (27.7%) developed HT. LASSO logistic regression revealed that infarct size, NIHSS score, diabetes mellitus, reperfusion therapy, left ventricular ejection fraction, and prehospital antihypertensive treatment were significant HT predictors. In the external validation cohort (*n* = 236), 61 patients (25.8%) developed HT. The nomogram exhibited strong predictive performance, with AUCs of 0.720 in the training set and 0.747 in the validation set.

**Conclusions:**

The proposed nomogram offers a practical tool for predicting HT risk in AIS patients with AF before anticoagulation reinitiation, potentially supporting informed clinical decision‐making, though further validation is required.

## Graphical abstract

A predictive nomogram for hemorrhagic transformation risk in acute ischemic stroke patients with atrial fibrillation was developed, using LASSO regression to identify key factors. The model aids clinical decision‐making prior to anticoagulation, potentially reducing bleeding complications and improving patient management.

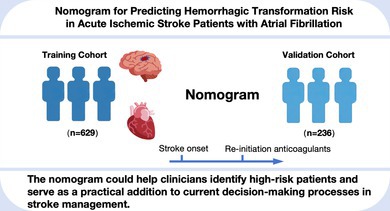

## 1 Introduction

About 10%–30% of all ischemic stroke cases are related to atrial fibrillation (AF) [1, 2, 3]. Compared with strokes of other subtypes, AF‐related ischemic stroke presents with distinct features, including multiple and bilateral cerebral infarcts, larger infarct volumes, and a greater propensity for hemorrhagic transformation (HT) [4, 5]. This condition, a common and severe neurological complication, is linked to higher mortality rates in ischemic stroke patients [6]. Because of the lack of consistency in its definition, the incidence of HT varies widely, ranging from 3% to 40%, across different studies [7, 8]. Even mild HT can lead to neurological deterioration and impact functional patient outcomes [9].

Anticoagulant therapy has been proven to be highly effective in preventing secondary stroke in patients with AF. Early anticoagulation treatment can prevent the recurrence of ischemic stroke but also increase the risk of HT [10]. Therefore, it is important to identify patients potentially at high risk of HT. Many clinical factors influence the development of HT, making it difficult to quickly evaluate the risk of this condition and the timing for initiating anticoagulation therapy. Previous studies have revealed that several factors (e.g., advanced age, hypertension, hyperglycemia, early infarction, reperfusion therapy, and stroke severity) are associated with HT in acute ischemic stroke (AIS) patients. However, few studies have specifically investigated HT among AIS patients with AF. Consequently, a dedicated exploration of HT in patients with AF is crucial.

Conventional multivariable logistic regression analyses, while widely used in the literature, fail to account for random errors and exhibit instability during the variable selection process. Therefore, the least absolute shrinkage and selection operator (LASSO) method has been adopted due to its robustness in managing high‐dimensional predictors [11, 12]. Given the increased risk of HT in stroke patients with AF, this study seeks to address the lack of research focusing on the time from stroke onset to the initiation of anticoagulation therapy. By retrospectively analyzing data from two cohorts, this study aimed to develop and validate a predictive model specifically for evaluating the risk of HT in AIS patients with AF prior to anticoagulation therapy. This model is intended to support clinicians in making informed, timely treatment decisions, potentially lowering the risk of severe bleeding complications and improving the management of stroke in patients with AF.

## 2 Methods

The study received approval from the medical ethics committees of Sun Yat‐Sen Memorial Hospital (2020‐KY‐005) and the First People's Hospital of Foshan ([2021] No. 38). The need for patient consent was waived because of the retrospective nature of the study.

### 2.1 Subjects

A nomogram was developed using data from patients at the Department of Neurology, First People's Hospital of Foshan, China. This retrospective study included patients diagnosed with AIS and AF from December 2016 to September 2022. Eligible patients who met the following criteria were recruited as the training cohort: (1) age ≥ 18 years; (2) admission within 3 days of ischemic stroke, regardless of prior anticoagulation or antiplatelet treatment; (3) radiological examination (magnetic resonance imaging, MRI, or computed tomography, CT) data supporting the diagnosis of AIS; and (4) self‐reported history of AF or detection via electrocardiography (ECG) following hospitalization. Patients with any of the following characteristics were excluded: (1) hemorrhage after initiating anticoagulation; (2) hemorrhage due to trauma, intracranial vascular malformation, neoplasm, or any other presumed cause; (3) poor‐quality brain imaging data; (4) no brain imaging data prior to anticoagulation therapy; or (5) other significant concomitant systemic diseases.

An independent cohort of patients was enrolled at Sun Yat‐sen Memorial Hospital, China, from November 2016 to April 2023, and those adhering to the same inclusion and exclusion criteria served as an external validation cohort.

### 2.2 Diagnosis and Treatment

A comprehensive search of the patient medical records was performed to identify AIS patients with AF, whose treatment data were also retrieved. AIS was defined by the presence of acute neurological deficits and confirmed by MRI or CT scans showing evidence of acute cerebral infarction. Infarct size was classified as minor, moderate, or major via a standardized visual rating scheme (Table S1) [13, 14, 15].

AF was detected via patient self‐reports, routine 12‐lead ECG, or 24‐h Holter ECG post hospitalization. All patients were assessed by experienced neurologists to determine the need for intravenous thrombolysis (IVT) or endovascular thrombectomy (EVT) and received standard secondary preventative treatment.

### 2.3 Data Collection and Parameter Definitions

The following demographic and clinical characteristics were recorded: age, sex, and National Institutes of Health Stroke Scale (NIHSS) score at initial presentation (trained neurologists used a standardized assessment form to collect data on functional status before stroke onset); comorbidities (e.g., hypertension, diabetes mellitus, and prior stroke or transient ischemic attack); and prehospital treatments, including antiplatelet, oral anticoagulation, antihypertensive, antihyperglycemic, and statin treatments (only daily medications were recorded).

The baseline variables obtained at admission included the levels of glycated hemoglobin (HbA1c), fasting serum cholesterol (total, high‐density lipoprotein cholesterol (HDL‐C), and low‐density lipoprotein cholesterol (LDL‐C)), and fasting serum triglycerides (TG); white blood cell count, platelet count, neutrophil count, and lymphocyte count; hemoglobin and fibrin levels; the international normalized ratio (INR); activated thromboplastin time (APTT); and D‐dimer and creatinine levels. Additionally, transthoracic echocardiography parameters, including left ventricular ejection fraction (LVEF, %) and left atrial diameter (LA, mm), were recorded within 6 months before or after admission.

The primary outcome was the occurrence of postinfarct HT before anticoagulation therapy. The occurrence of HT was assessed via imaging performed at several key points: upon admission, 24 h after reperfusion therapy (if received), at the time of clinical deterioration (if observed), and before anticoagulation initiation. HT was categorized on the basis of the Heidelberg Bleeding Classification [16] by two experienced radiologists who evaluated the imaging examinations independently; a third radiologist provided a final judgment in cases of disagreement.

### 2.4 Statistical Analysis

Potential predictors of HT were identified from the published literature (e.g., advanced age, male sex, medical comorbidity, stroke severity at presentation, hypertension, hyperglycemia, early infarction signs on brain imaging, low platelet counts, reperfusion therapy, and antithrombotic drugs) [17, 18, 19, 20, 21, 22], clinical experience, and the results of routine tests of patients with AIS. The normality of continuous variables was assessed using the Shapiro–Wilk test, and none of the variables conformed to a normal distribution. Data were described using medians (interquartile ranges, IQR) for continuous variables and frequencies with percentages for categorical variables. The Mann–Whitney *U* test was used for comparison between groups of continuous variables, and Chi‐square or Fisher's exact tests were used for categorical variables, as appropriate. Multiple imputation was performed for missing values prior to the regression analyses.

The LASSO logistic regression algorithm was applied to the training set to screen for risk factors for HT in AIS patients with AF. LASSO regression was chosen because of its ability to handle multicollinearity and identify variables that contribute most to the outcome. Significant predictors identified by LASSO regression were then entered into multivariable logistic regression models to establish the final prediction models. The continuous net reclassification improvement (NRI) and integrated discrimination improvement (IDI) values were used to determine the reclassification power of different models. A predictive nomogram was generated on the basis of the model that showed the greatest performance among the different models. Regression coefficients and odds ratios (ORs) with 95% confidence intervals (CIs) for each variable were calculated. Multicollinearity was evaluated via the variance inflation factor (VIF), with an arithmetic square root of VIF ≤ 2 indicating noncollinearity.

The discriminative ability of the nomogram was evaluated with the area under the receiver operating characteristic curve (AUC) in the training set. Calibration was assessed with the Hosmer–Lemeshow test and a calibration plot, which was generated using a bootstrap method with 1000 resamples. To validate the nomogram, AUC, Hosmer–Lemeshow test, and calibration curve plotting were similarly applied to the external validation set.

Decision curve analysis (DCA) was used to quantify the nomogram's clinical utility across different threshold probabilities in the training and validation sets [23]. Statistical analyses were conducted with R software (version 4.3.1, http://www.r‐project.org/). LASSO logistic regression was performed with the “glmnet” package, VIF values were measured with the “car” package, the NRI and IDI were calculated with the “PredictABEL” package, the nomogram was plotted with the “nomogramFormula” package, ROC curves were constructed with the “pROC” package, and calibration plots and DCA were created with the “rmda” package. A *p* value < 0.05 was considered to indicate statistical significance.

## 3 Results

### 3.1 Patient Demographics and Clinical Data

A total of 629 patients meeting the inclusion and exclusion criteria formed the training cohort, 174 (27.7%) of whom experienced postinfarct HT before anticoagulant therapy. The external validation cohort included 236 AIS patients with AF, with 61 (25.8%) having HT. The demographic, clinical, and radiological characteristics and laboratory findings of both cohorts are summarized in Table 1. Significant differences between the two cohorts were observed in the proportions of patients receiving antiplatelet, anticoagulation, and statin treatment before the onset of AIS, as well as in the results of several serum tests, LA, and LVEF.

**TABLE 1:** Baseline characteristics of the patients enrolled.

	The training set *N* = 629	The external validation set *N* = 236	*p*.overall
Sex			
Male	345 (54.8%)	140 (59.3%)	0.27
Female	284 (45.2%)	96 (40.7%)	
Age (y)	73 (66–79)	74 (65–81)	0.191
Medical history			
Hypertension	401 (63.8%)	160 (67.8%)	0.303
Diabetes mellitus	142 (22.6%)	67 (28.4%)	0.091
Coronary heart disease	101 (16.1%)	47 (19.9%)	0.215
Prior stroke/TIA	149 (23.7%)	43 (18.2%)	0.103
Valvular disease of the heart	68 (10.8%)	22 (9.32%)	0.607
Antihypertensive treatment	235 (37.4%)	105 (44.5%)	0.067
Antihyperglycemic treatment	88 (14.0%)	46 (19.5%)	0.059
Antiplatelet treatment	27 (4.29%)	24 (10.2%)	0.002
Oral anticoagulant treatment	103 (16.4%)	24 (10.2%)	0.029
Statin treatment	14 (2.23%)	15 (6.36%)	0.005
Baseline data			
NIHSS	7 (3–13)	6 (3–11)	0.308
Reperfusion therapy	155 (24.6%)	69 (29.2%)	0.198
Hb (g/L)	134 (122–146)	131 (115–144)	0.04
Lym (×10^9^/L)	1.33 (0.98–1.78)	1.39 (1.02–1.74)	0.667
Neu (×10^9^/L)	6.24 (4.59–8.30)	5.83 (4.38–8.16)	0.202
Plt (×10^9^/L)	213 (172–258)	208 (169–259)	0.678
RBC (×10^12^/L)	4.45 (4.08–4.92)	4.39 (3.88–4.84)	0.06
WBC (×10^9^/L)	8.50 (6.84–10.6)	8.22 (6.58–10.3)	0.26
HDL‐C (mmol/L)	1.08 (0.92–1.31)	1.06 (0.88–1.26)	0.046
LDL‐C (mmol/L)	2.42 (1.91–3.09)	2.60 (2.12–3.35)	0.001
TG (mmol/L)	1.08 (0.84–1.44)	1.09 (0.88–1.49)	0.376
TC (mmol/L)	4.16 (3.49–4.81)	4.22 (3.44–5.16)	0.226
Cre (μmol/L)	76.0 (60.0–92.0)	86.0 (73.0–100)	< 0.001
APTT (s)	27.2 (25.1–30.2)	26.6 (24.5–30.1)	0.096
FIB (g/L)	3.00 (2.43–3.67)	3.19 (2.58–3.96)	0.03
INR	1.10 (1.02–1.19)	1.06 (1.00–1.16)	0.002
D‐dimer (μg/mL)	0.11 (0.07–0.70)	1.17 (0.55–2.95)	< 0.001
HbA1c (%)	6.00 (5.60–6.55)	5.90 (5.50–6.60)	0.097
LA (mm)	42 (38–47)	40 (37–45)	0.034
LVEF (%)	61 (57–66)	64 (60–68)	< 0.001
Location			
ACI	491 (78.4%)	171 (74.0%)	0.22
PCI	114 (18.2%)	54 (23.4%)	
ACI and PCI	21 (3.35%)	6 (2.60%)	
Infarct size			
Minor or moderate	312 (49.8%)	128 (55.4%)	0.17
Major	314 (50.2%)	103 (44.6%)	
Outcome			
HT	174 (27.7%)	61 (25.8%)	0.654
Heidelberg bleeding classification			0.657
Class 1	121 (69.1%)	41 (67.2%)	
Class 2	21 (12.0%)	10 (16.4%)	
Class 3	33 (18.9%)	10 (16.4%)	

*Note:* Data are *n* (%), or median (IQR).

Abbreviations: ACI, anterior circulation infarct; APTT, activated partial thromboplastin time; Cre, creatinine; FIB, fibrin; Hb, hemoglobin; HbA1c, glycosylated hemoglobin; HDL‐C, high‐density lipoprotein cholesterol; INR, international normalized ratio; LA, left atrial diameter; LDL‐C, low‐density lipoprotein cholesterol; LVEF, left ventricular ejection fraction; Lym, lymphocyte; Neu, neutrophils; NIHSS, National Institutes Stroke Scale; PCI, posterior circulation infarct.Plt, platelet; RBC, red blood cell; TC, total cholesterol; TG, triglyceride; TIA, transient ischemic attack; WBC, white blood cell.

**FIGURE 1:**
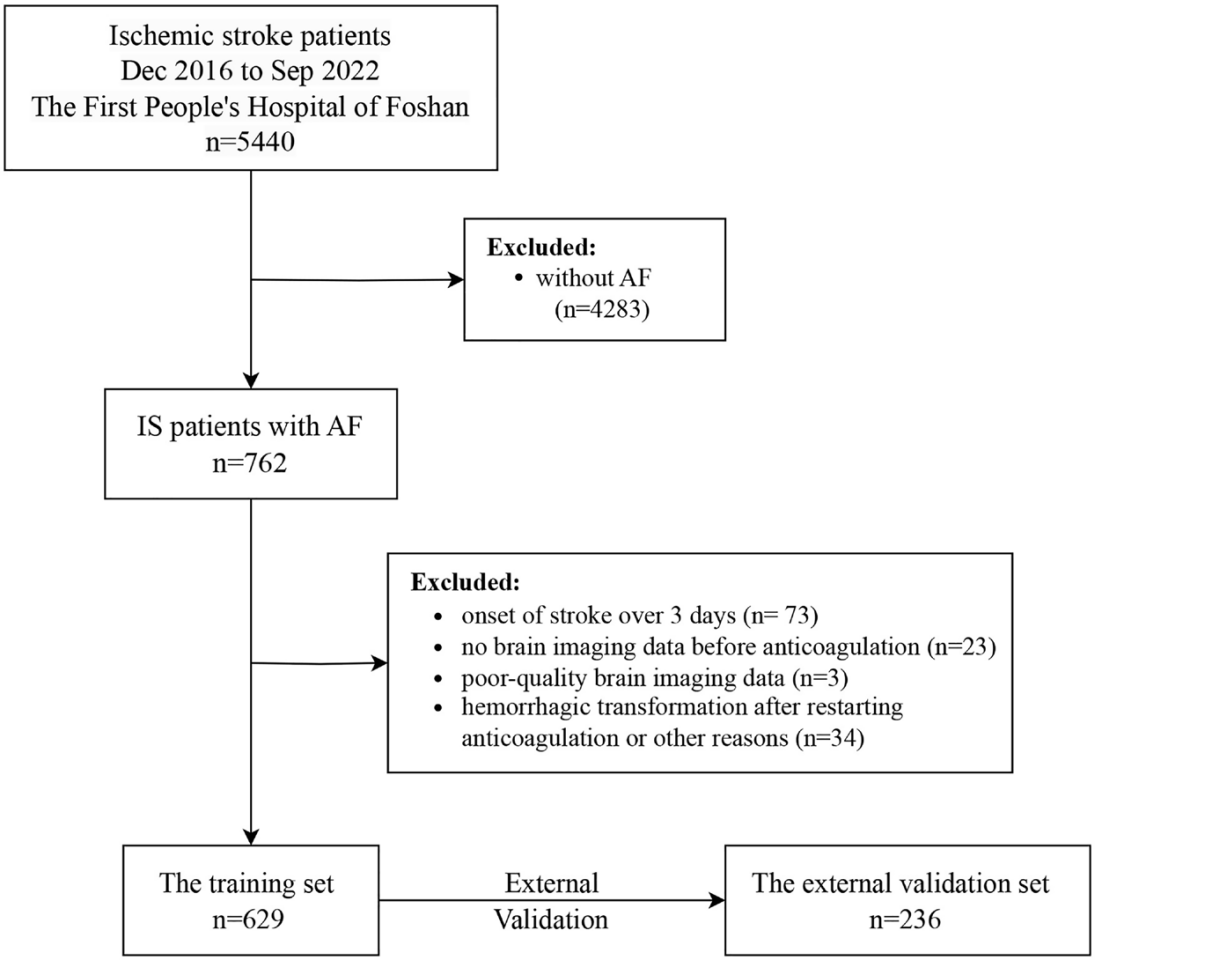
Study flowchart. AF, atrial fibrillation; IS, ischemic stroke.

According to the Heidelberg Bleeding Classification, the training cohort included 121 participants (69.1%) in Class 1, 21 participants (12%) in Class 2, and 33 participants (18.9%) in Class 3. Comparable proportions were observed in the external validation set. The study flowchart is presented in Figure 1.

### 3.2 Prediction Model Built According to the Results of LASSO Logistic Regression

The results of the univariable and multivariable analyses are presented in Table 2. Univariable analysis revealed that diabetes mellitus, reperfusion therapy, LVEF, major infarct size, HDL‐C level, and the presence of anterior circulation infarction (ACI) were associated with HT (*p* < 0.05). A higher level of TG was identified as a protective factor against HT (*p* < 0.05).

**TABLE 2:** Univariable and multivariable adjusted analyses.

Variables	Univariable analysis		Multivariable adjusted analysis	
	OR (95% CI)	*p*	OR (95% CI)	*p*
Diabetes mellitus (yes vs. no)	2.74 (1.30–5.82)	0.0080*	1.67 (1.08–2.58)	0.021*
Antihypertensive treatment (yes vs. no)	0.75 (0.44–1.27)	0.29	0.65 (0.43–0.98)	0.039*
NIHSS	1.12 (0.82–1.53)	0.46	1.01 (0.98–1.04)	0.36
Reperfusion therapy (yes vs. no)	2.05 (1.27–3.31)	0.0032**	1.91 (1.25–2.92)	0.0028**
HDL‐C (mmol/L)	6.42 (1.78–27.54)	0.0074**		
TG (mmol/L)	0.22 (0.06–0.64)	0.011*		
LVEF (%)	1.03 (1.00–1.05)	0.024*	1.02 (1.00–1.05)	0.029*
Location (PCI vs. ACI)	0.49 (0.28–0.88)	0.016*		
(ACI and PCI vs. ACI)	2.12 (0.79–5.70)	0.14		
Infarct size (major vs. minor/moderate)	3.87 (2.48–6.13)	< 0.0001**	3.39 (2.25–5.19)	< 0.0001**

Abbreviations: ACI, anterior circulation infarct; CI, confidence interval; HDL‐C, high‐density lipoprotein cholesterol; LVEF, left ventricular ejection fraction; NIHSS, National Institutes Stroke Scale; PCI, posterior circulation infarct; TG, triglyceride.

*p < 0.05, **p < 0.01.

Model 1 included six variables selected by LASSO regression for multivariable logistic regression: prehospital antihypertensive treatment (OR, 0.65; 95% CI, 0.43–0.98; *p* = 0.039), diabetes mellitus (OR, 1.67; 95% CI, 1.08–2.58; *p* = 0.021), reperfusion therapy (OR, 1.91; 95% CI, 1.25–2.92; *p* = 0.0028), major infarct size (OR, 3.39; 95% CI, 2.25–5.19; *p* < 0.0001), NIHSS score (OR, 1.01; 95% CI, 0.98–1.04; *p* = 0.36), and LVEF (OR, 1.02; 95% CI, 1.00–1.05; *p* = 0.029) (Table 2).

**FIGURE 2:**
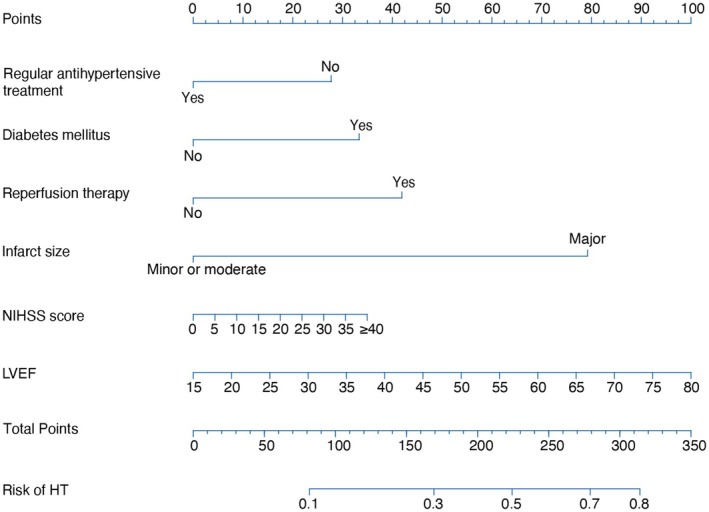
Nomogram for predicting HT in AIS patients with AF. Points are assigned on the basis of regular antihypertensive treatment, diabetes mellitus, reperfusion therapy, infarct size, NIHSS score, and LVEF by drawing a line upward from the corresponding values to the “Points” line. The sum of these six points, plotted on the “Total points” line, corresponds to the prediction of HT risk. HT, hemorrhagic transformation; LEVF, left ventricular ejection fraction; NIHSS, National Institutes of Health Stroke Scale.

Model 2 was constructed by excluding the NIHSS score (due to *p* = 0.36) from Model 1. This model demonstrated significantly lower reclassification power than Model 1 in the external validation set (continuous NRI [95% CI]: −0.4335 [−0.7183 – −0.1488], *p* = 0.0029; IDI [95% CI]: −0.0192 [−0.037 to −0.0013], *p* = 0.0350; Table S2). Several previous studies have identified the NIHSS score as an independent predictor of postinfarction HT. Therefore, the NIHSS score was retained in the final model, and a nomogram was developed based on this final model (Figure 2). No significant covariance was found among the variables in the model.

### 3.3 Calibration and Clinical Application of the Nomogram

**FIGURE 3:**
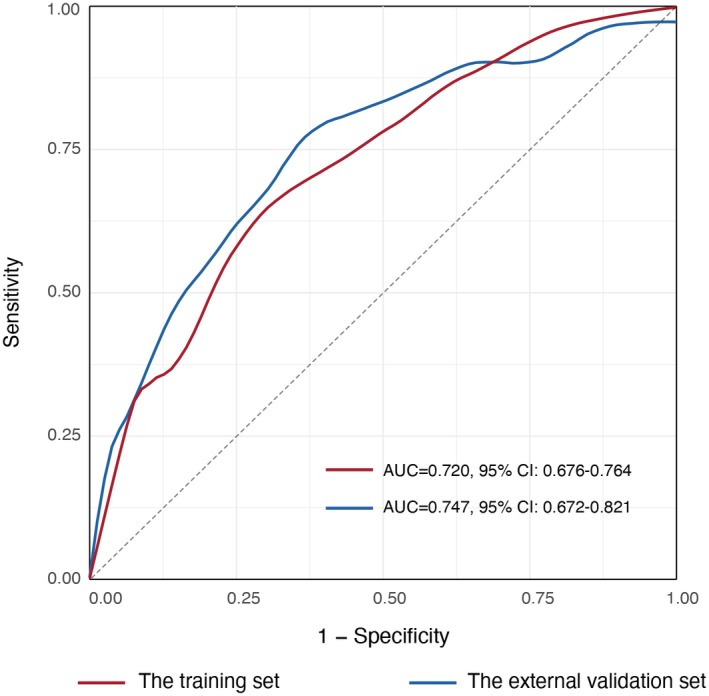
ROC curves of the nomogram. Receiver operating characteristic curves were used to assess the discriminative ability of the nomogram in the training and external validation sets.

The nomogram demonstrated good discrimination, with an AUC of 0.720 (95% CI, 0.676–0.764) in the training set and 0.747 (95% CI, 0.672–0.821) in the external validation set (Figure 3). The Hosmer–Lemeshow tests yielded nonsignificant results in both the training and external validation sets (*p* = 0.37 and *p* = 0.47, respectively), indicating acceptable goodness‐of‐fit between the predicted probabilities and the actual occurrence of HT.

**FIGURE 4:**
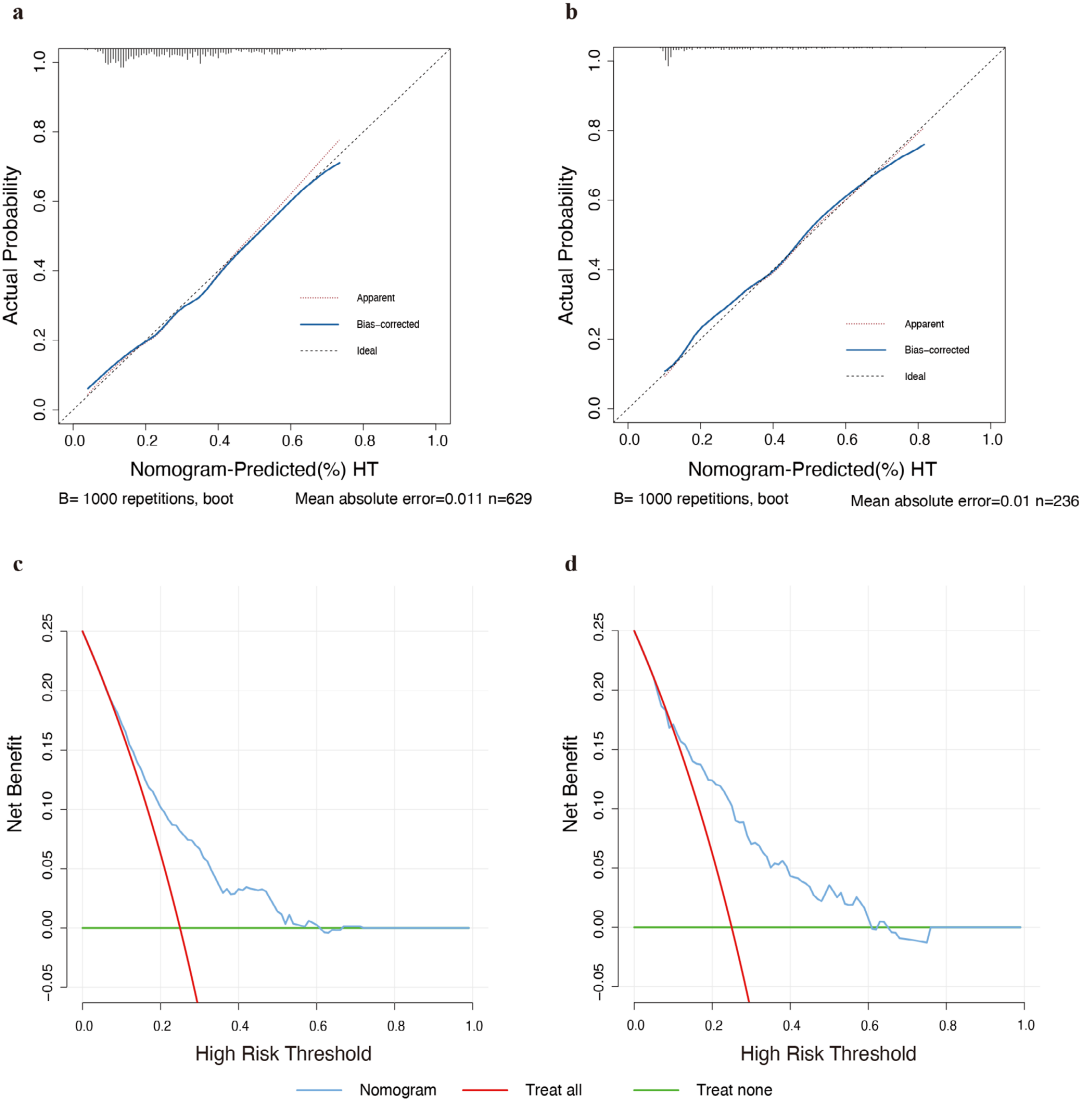
Calibration plots and decision curve analysis plots of the nomogram. Calibration plots for the nomogram‐predicted probability of hemorrhagic transformation in the training (a) and external validation sets (b). Decision curve analysis plots of the nomogram in the training (c) and external validation sets (d). The green lines depict the net benefit of a strategy of treating no patients. The red lines depict the net benefit of a strategy of treating all patients. The blue lines represent the net benefit of nomogram‐guided clinical decision‐making, with lines closer to the top right of the figure indicating a greater net benefit. HT, hemorrhagic transformation.

Calibration plots revealed that the apparent line and bias‐corrected line deviated only slightly from the ideal line, suggesting high accuracy in predicting the actual probability of HT in both the training set (Figure 4a) and the external validation set (Figure 4b). DCA was conducted to estimate the net benefit of using the nomogram for clinical decision‐making. The results of DCA indicated that employing the nomogram for guiding clinical decisions was superior to the “treat all” or “treat none” strategies when the predicted probabilities of HT ranged from 7% to 61% in the training set (Figure 4c) and from 10% to 61% in the external validation set (Figure 4d).

## 4 Discussion

HT is a common complication of AIS and is more prevalent in patients with AF. To our knowledge, this study is the first to develop a nomogram that evaluates the risk factors for postinfarct HT in AIS patients with AF prior to anticoagulation therapy. This novel predictive tool was externally validated, showing good discrimination and calibration. The results revealed items that should be prioritized for assessment in clinical practice: NIHSS score at admission, diabetes mellitus status, prehospital antihypertensive treatment, LVEF, infarct size, and reperfusion therapy. If a patient is predicted to have an increased risk of HT according to the nomogram, delaying anticoagulant treatment should be considered.

In our study, the incidence of HT in AIS patients with AF before anticoagulation was restarted was approximately 27%. Several established risk factors, such as higher NIHSS scores, diabetes mellitus, and reperfusion therapy, are related to HT [6, 21]. Patients with diabetes mellitus are more prone to HT, which is consistent with previous findings [24, 25]; moreover, this risk extends to diabetic patients receiving reperfusion therapy [26]. Conversely, prehospital antihypertensive treatment before stroke reduces the risk of HT. Studies have shown that lowering prestroke blood pressure can decrease the risk of HT [27, 28]. Both hypertension and hyperglycemia are linked to disruption of the blood–brain barrier (BBB), which may lead to HT through mechanical changes or acute endothelial and inflammatory activation [29]. Additionally, postinfarct HT typically occurs several days to 1 or 2 weeks after stroke onset [30]. Unlike previous studies that primarily focused on admission glucose and blood pressure levels, which can be influenced by stress, our findings emphasize the importance of considering a patient's prestroke history of diabetes mellitus or antihypertensive treatment before restarting anticoagulation therapy.

The incorporation of radiologic signatures, in addition to clinical features, is essential for developing more comprehensive models. A standardized visual rating scheme was employed to categorize infarcts into minor, moderate, and major sizes. A major infarct size emerged as the strongest predictor of HT in the nomogram, with an OR of 3.39. This scheme is easier to use than other imaging markers (e.g., poor collateral circulation and lesion‐side calcification volume), making it highly favorable for patient differentiation.

The novel finding of this study is that HT was associated with supranormal left ventricular ejection fraction (snLVEF). A previous study revealed that all‐cause mortality had a U‐shaped relationship with LVEF, with the risk reaching a minimum at an LVEF of 60%–65% and a hazard ratio (HR) for death of 1.71 when the LVEF was ≥ 70% [31]. However, no previous study has demonstrated a correlation between LVEF and HT. Our study revealed an OR of 1.53 (95% CI, 0.93–2.46; *p* = 0.087) for HT in patients with an LVEF > 70% with respect to those with an LVEF of 40%–70%, but the result was not statistically significant. This phenomenon was even more noticeable in the external validation set, with an OR of 2.51 (95% CI, 1.0076–6.0826; *p* = 0.043). After adjusting for age, sex, and LA, the OR was 2.57 (95% CI, 1.0058–6.4341; *p* = 0.044). Despite this potentially significant correlation between the occurrence of HT and LVEF, their relationship requires further exploration.

This phenomenon may be attributed to impaired cerebrovascular autoregulation in AF patients. The alterations in cerebral perfusion induced by a cardiac arrhythmia, characterized by rapid and irregular beating of the atrial chambers of the heart, may further exacerbate this effect. Autoregulation of cerebral blood flow (CBF) is diminished in patients with AF relative to that of patients without AF. In one study, two coupled lumped‐parameter models (systemic and cerebrovascular circulations) showed that cerebral hemodynamic variables induced by AF were more variable than sinus rhythm [32]—this variability in CBF at the arterial level results in severe cerebral hemodynamic events [33, 34]. Researchers have reported greater changes in the mean blood flow velocity in the middle cerebral artery in AF patients than in those without AF, indicating impaired autoregulation [35, 36]. For AF patients with snLVEF, impaired cerebrovascular autoregulation increases the vulnerability of CBF to changes in response to fluctuations in cardiac output. This, combined with factors such as disruption of the BBB, may increase the likelihood of postinfarct HT.

This study is the first to develop a model for predicting the risk of postinfarct HT in AIS patients with AF before anticoagulation therapy is restarted. A key strength is the use of advanced statistical methods such as LASSO regression to identify significant predictors, which helps prevent overfitting and multicollinearity, ensuring that the final nomogram is robust. Clinically relevant variables, such as diabetes mellitus, reperfusion therapy, infarct size, and LVEF increase the model's potential for real‐world applicability. The model was validated in an independent cohort, supporting its reliability and generalizability across clinical settings. The nomogram represents a step toward personalized medicine by identifying individuals at high risk for HT and can help assess the risk of postinfarct hemorrhage before anticoagulation therapy is started. Future prospective studies should explore the optimal timing for initiating anticoagulant therapy in different risk groups.

Our study has several limitations. First, the retrospective nature of imaging data collection may have introduced selection bias, as patients clinically suspected to be at high risk for HT were more likely to be included, whereas patients with poor conditions may have been excluded owing to inadequate imaging quality. Future prospective studies are necessary to further validate our results. Second, both CT and MRI were used to detect HT. Although susceptibility‐weighted imaging (SWI) is more sensitive than CT in detecting minor bleeding, some patients only underwent repeat CT scans, which could have led to missed diagnoses of minor bleeding. Additionally, we did not classify patients on the basis of the type of AF. Future research should explore differences in HT among patients with paroxysmal, persistent, and permanent AF. Furthermore, details on treatments that may influence HT, such as antiplatelet therapies after admission and the duration between onset and reperfusion therapy, were not included.

## 5 Conclusions

The nomogram demonstrated promising predictive accuracy and clinical utility in the two cohorts studied. By incorporating multiple clinical and imaging‐based predictors, our model provides a quantitative tool that may assist clinicians in identifying high‐risk patients and inform decisions regarding the timing of anticoagulation reinitiation. Given the retrospective design, further prospective studies are warranted to evaluate the model's real‐world applicability and impact across diverse clinical settings. The implementation of this tool may potentially contribute to reducing HT‐related complications and improving outcomes in AIS patients with AF.

## Author Contributions

J.C., M.B., and Y.T. researched the literature and conceived the study. J.C., C.Z., F.Z., D.P., Y.X., Y.C., and Y.T. provided administrative, technical, or material support. J.C. and M.B. participated in material preparation, data collection, and analysis. M.B. wrote the original draft of the manuscript. All the authors reviewed and edited the manuscript and approved the final version of the manuscript.

## Ethics Statement

The study was conducted in accordance with the Declaration of Helsinki and was approved by the medical ethics committees of Sun Yat‐Sen Memorial Hospital (no. 2020‐KY‐005) on February 27th, 2020, and First People's Hospital of Foshan ([2021] No. 38) on February 4th, 2021.

## Consent

Patient consent was waived because of the retrospective nature of the study.

## Conflicts of Interest

The authors declare no conflicts of interest.

## Supporting information


Tables S1–S2.

## Data Availability

The data supporting this study's findings are available from the corresponding author upon reasonable request.
