# Supplementary material for: Nomogram for Predicting Hemorrhagic Transformation Risk in Acute Ischemic Stroke Patients With Atrial Fibrillation
Source: CNS Neurosci Ther. 2025 Apr 26;31(4):e70402. doi: 10.1111/cns.70402 (PMC12032400; doi:10.1111/cns.70402)
Supplement: Supplementary file 1 — Tables S1–S2. [file CNS-31-e70402-s001.docx]

**Supplementary Table S1. Infarct size classification and definition.**

| Infarct size classification | Definition |
| --- | --- |
| Minor | **small <1.5 cm lesion:** in anterior or posterior circulation |
| Moderate | **medium lesion:** in a cortical superficial branch of ACA, MCA, or PCA, or deep branch of MCA or PCA, or in internal border-zone territories; |
| Major | **large anterior lesion:** complete territory of ACA or MCA; two branches of MCA; or >1 artery territory; |
|  | **large posterior lesion:** involving brain stem, cerebellum >1.5 cm, or complete territory of PCA together with border-zone territories. |

Abbreviation: ACA, anterior cerebral artery; MCA, middle cerebral artery; PCA, posterior cerebral artery.

**Supplementary Table S2. Comparison of Model 1 and Model 2.**

|  | The training set | | | The external validation set | | |
| --- | --- | --- | --- | --- | --- | --- |
|  | Model 1 | Model 2 | p-Value | Model 1 | Model 2 | p-Value |
| Continuous NRI [95% CI] | Ref | -0.2855[-0.4587- -0.1124] | 0.00123 | Ref | -0.4335 [-0.7183 - -0.1488] | 0.00285 |
| IDI [95% CI] | Ref | -0.0008[-0.0046 - 0.0029] | 0.65785 | Ref | -0.0192[-0.037--0.0013] | 0.03504 |

Model 1 included six variables, namely pre-hospital antihypertensive treatment, diabetes mellitus, reperfusion therapy, infarct size, NIHSS score, and LVEF. Model 2 incorporated five variables: pre-hospital regular antihypertensive treatment, diabetes mellitus, reperfusion therapy, infarct size, and LVEF.

Abbreviations: NRI, net reclassification improvement; IDI, integrated discrimination improvement; Ref, reference.
